
JOURNAL INFORMATION
==============================
NLM Title Abbreviation: South Med Rec
NLM Journal ID: 0027317

The Southern Medical Record

Publisher: Biomedical Journal Digitization Project

ARTICLE INFORMATION
==============================
PMCID: PMC9089564
PMID: 36023166
Article version: 1
Subjects: Editorial and Miscellaneous

Give Us Credit

Print publication date: 1879 Mar 20
Volume: 9
Issue: 3
First page: 120
Last page: 120
License: This work is free of known copyright restrictions. For more information, please see PMC Back Issue Digitization.
License URL: https://creativecommons.org/publicdomain/mark/1.0/

==============================

### *GIVE US CREDIT.*

A "*Western*" journal—yea, two journals out that way—copied from our pages without giving us credit therefor. Brethren, it was perhaps accidental, and the articles used of but little value; and it may be that the best of us make these oversights; in "*Brief,*" however, we would say, let us not forget these little courtesies.

☞SUBSCRIBERS in arrears are requested to remit their subscriptions at once.

### *BUFFALO LITHIA WATER.*

See advertisement of this celebrated water, in our present issue.

RECIPTS.—Being pressed for space, we have omitted receipts of subscriptions for the past month. They will appear in our next.

### SPECIAL NOTICES.

OUR line now consists of Fluid Extracts, Sugar-coated Pills and Granules, Solid Extracts, Concentrations, Powdered Solid Extracts, Specific Tinctures, Medicinal Elixirs, Wines and Syrups, Collodions, Cerates, Confections, Lozenges, &c., and of Fluid Extracts and Pills we are probably the *largest Manufacturers in the United States.* We make a specialty of New Preparations, and have introduced many such to the profession. Send stamp for Descriptive Circular, giving botanical history and therapeutical application of fifty new remedies.

PARKE, DAVIS & CO.,
Manufacturing Chemists, DETROIT.

**INGLUVIN.**—Since the discovery and introduction of Pepsin into medical practice, many years ago, its use has continued as a remedy with variable results. Recently, the reliable firm of W. R. WARNER & Co., of Philadelphia, have discovered and prepared a preparation in the form of powder, made from the Ventriculus Callo Gallenaceus (the gizzard of the domestic fowl), which they denominate "INGLUVIN," from *Ingluvies*, the craw. Reports of its beneficial effects from the highest authorities of the medical profession, and no less than Professor Wallace, of "Jefferson Medical College," establishes its importance as a remedy far beyond Pepsin and its preparations. It is presented in similar doses and combinations. It has a specific action in cases of sickness in pregnancy.
